# The International Polycap Study-3 (TIPS-3): Design, baseline characteristics and challenges in conduct

**DOI:** 10.1016/j.ahj.2018.07.012

**Published:** 2018-12

**Authors:** Philip Joseph, Prem Pais, Antonio L Dans, Jackie Bosch, Denis Xavier, Patricio Lopez-Jaramillo, Khalid Yusoff, Anwar Santoso, Shamim Talukder, Habib Gamra, Karen Yeates, Paul Camacho Lopez, Jessica Tyrwhitt, Peggy Gao, Koon Teo, Salim Yusuf, on behalf of the TIPS-3 Investigators

**Affiliations:** a Population Health Research Institute, Hamilton Health Sciences and McMaster University, Hamilton, Ontario, Canada; b St. John's Medical College, Bangalore, India; c College of Medicine, University of the Philippines, Manila, Philippines; d School of Rehabilitation Science, McMaster University, Hamilton, Ontario, Canada; e Research Institute and Clinic of Metabolic Syndrome and Diabetes, Fundacion Oftalmologica de Santander FOSCAL, Universidad de Santander UDES, Bucaramanga, Colombia; f UiTM Selayang, Selangor and UCSI University, Cheras, Kuala Lumpur, Malaysia; g Universitas Indonesia and Department of Cardiology - Vascular Medicine, National Cardiovascular Centre, Harapan Kita Hospital, Jakarta, Indonesia; h Eminence, Dhaka, Bangladesh; i Fattouma Bourguiba University Hospital and University of Monastir, Tunisia; j Department of Medicine, Queen's University, Kingston, Ontario, Canada

## Abstract

**Background:**

It is hypothesized that in individuals without clinical cardiovascular disease (CVD), but at increased CVD risk, a 50% to 60% reduction in CVD risk could be achieved using fixed dose combination (FDC) therapy (usually comprised of multiple blood-pressure agents and a statin [with or without aspirin]) in a single “polypill”. However, the impact of a polypill in preventing clinical CV events has not been evaluated in a large randomized controlled trial.

**Methods:**

TIPS-3 is a 2x2x2 factorial randomized controlled trial that will examine the effect of a FDC polypill on major CV outcomes in a primary prevention population. This study aims to determine whether the Polycap (comprised of atenolol, ramipril, hydrochlorothiazide, and a statin) reduces CV events in persons without a history of CVD, but who are at least at intermediate CVD risk. Additional interventions in the factorial design of the study will compare the effect of (1) aspirin versus placebo on CV events (and cancer), (2) vitamin D versus placebo on the risk of fractures, and (3) the combined effect of aspirin and the Polycap on CV events.

**Results:**

The study has randomized 5713 participants across 9 countries. Mean age of the study population is 63.9 years, and 53% are female. Mean INTERHEART risk score is 16.8, which is consistent with a study population at intermediate CVD risk.

**Conclusion:**

Results of the TIP-3 study will be key to determining the appropriateness of FDC therapy as a strategy in the global prevention of CVD.

## Introduction and rationale of the TIPS-3

With 80% of cardiovascular disease (CVD) cases now occurring in low- and middle-income countries (LICs and MICs), there is a growing need to implement CVD preventive strategies that are highly impactful, low cost, and can be adopted across a range of health resource settings. An immediately impactful strategy is to modify major risk factors for CVD development using combinations of proven, safe, widely available and inexpensive drugs. This approach has been the basis for the development of fixed dose combination (FDC) therapy or “the polypill concept” for the prevention of CVD.

Studies comparing the effects of a FDC pill (usually containing two or three blood pressure agents and a statin) on risk factor levels have shown that significant reductions in blood pressure and cholesterol levels can be achieved, with better adherence compared to usual care.^1^ Furthermore, the extent of blood pressure and cholesterol lowering achieved could translate to reductions in CVD risk ranging from 50–60%.^1^ However, studies that directly examine clinical outcomes with a FDC pill are lacking, and even a meta-analysis of existing trials had too few events to provide a reliable estimate of the benefits of a polypill on CVD.^2^ The Heart Outcomes Prevention Evaluation (HOPE)-3 placebo-controlled randomized controlled trial (RCT) tested a “strategy” of FDC blood pressure and cholesterol lowering therapy with candesartan plus hydrochlorothiazide (16/12.5 mg/day) in addition to rosuvastatin (10 mg/day) (as separate agents), and observed that their combination reduced major cardiovascular events by 29% in persons at intermediate risk for developing CVD, with a 40% relative risk reduction (RRR) in those with elevated blood pressure.3., 4. Even larger reductions in CVD risk may be achievable with more intensive regimens, but data are needed that directly examine the clinical benefits and tolerance of such a strategy using a single FDC pill, which is the focus of this study.

TIPS-3 is a 2x2x2 factorial, RCT that will examine the effect of a FDC polypill on CVD outcomes in a primary prevention population. This study aims to determine whether the Polycap (comprised of atenolol, ramipril, hydrochlorothiazide, and a statin) reduces CV events in persons without a history of CVD, but who are at least at intermediate CVD risk. Additional interventions evaluated in the factorial design of the study compare the effect of (1) aspirin versus placebo on CV events (and cancer), (2) vitamin D versus placebo on the risk of fractures, and (3) the combination of aspirin and the Polycap on CV events (versus double-placebo).

Recruitment of TIPS-3 began in 2012 and we initially planned to enroll 5000 participants over a 2-year period. Despite the study testing a polypill comprised of commonly available and well tolerated medications, unanticipated regulatory challenges and restrictions on drug importation occurred in several countries. This contributed to substantial delays to study initiation and slowed down study enrolment, necessitating a substantial prolongation of the duration of the trial. Consequently, the enrollment of participants took 5 years (instead of 2 years), and interruptions in drug resupply during follow-up have led to higher than expected study drug discontinuation rates. To preserve statistical power this has required a larger sample size (N = 5713), and will require longer participant follow-up (i.e. until 2019–2020 as opposed to the original planned study end of Dec 2017). This article summarizes the design of TIPS-3, and baseline characteristics of the enrolled participants. Also, given that there is an increasing need for trials that include LICs and MICs, we examined the impact of regulatory factors on study recruitment and other aspects of study conduct.

## Methods

### Study objectives, design, and interventions

TIPS-3 is a double-blind, randomized, placebo controlled trial. Using a 2x2x2 factorial design, first we are testing the effect of the Polycap (comprised of atenolol 100 mg/daily, ramipril 10 mg/daily, hydrochlorothiazide 25 mg/daily, and simvastatin 40 mg/daily) versus placebo on major CV events. In the second factorial, we are testing the effect of aspirin 75 mg/day versus placebo on major CV events (and cancer). In the third factorial, we are testing the effect of vitamin D 60,000 IU given monthly on the risk of fractures compared to placebo.

**Figure 1:**
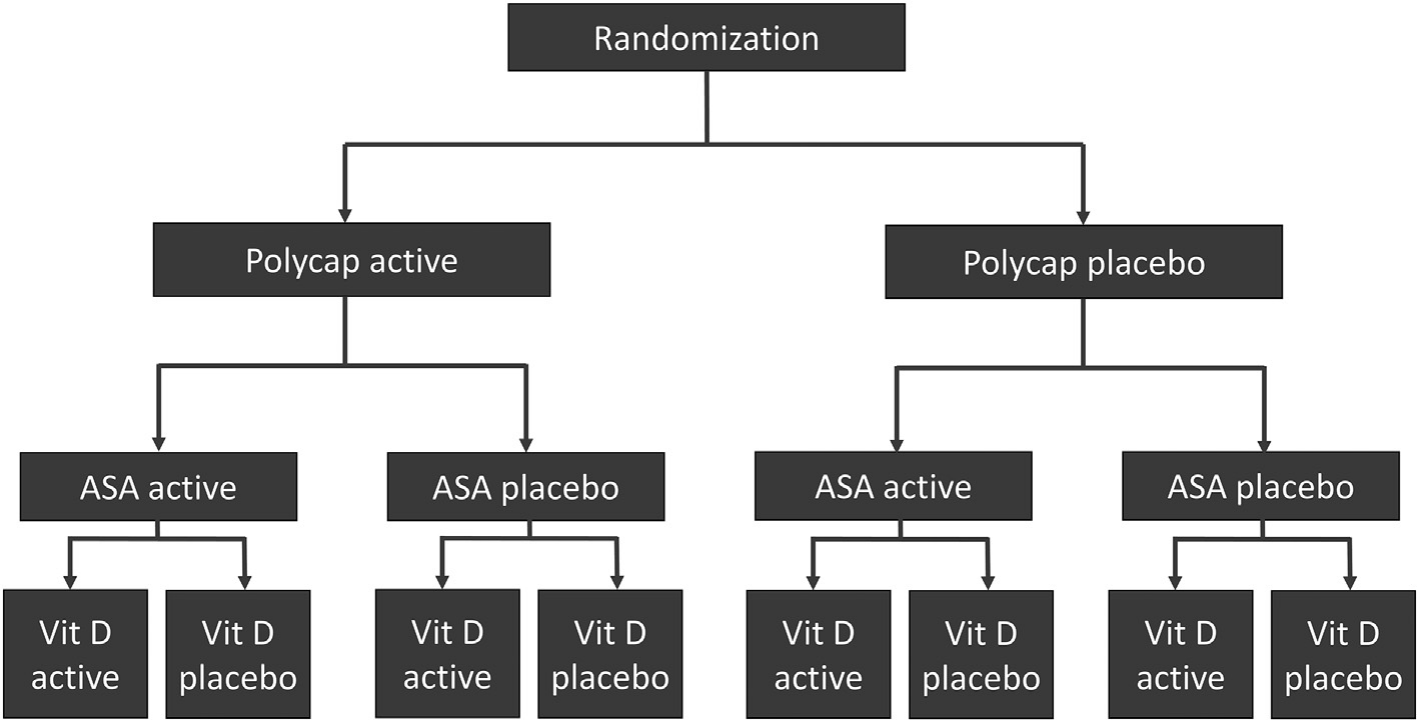
2x2x2 factorial study design of TIPS-3. Vit D = vitamin D.

The purpose of the factorial design is to assess the effects of each of the three distinct treatments within one efficient design (using 3 separate randomizations) in the same study population (see Figure 1). Therefore, participants randomized to the Polycap will be compared to the participants randomized to its placebo; participants allocated to aspirin will be compared to those on placebo for aspirin; and participants allocated to vitamin D will be compared participants allocated to placebo for vitamin D. The net clinical benefit of aspirin in primary prevention remains unclear, and after debating whether the polypill we are evaluating should include ASA, we ultimately chose to randomize participants to ASA or its placebo in a factorial design to gain information on the effects of ASA alone.5., 6. However, the combined effect of the Polycap with aspirin (i.e. the double-treatment group) will also be compared to the double-placebo group as part of our pre-specified analysis. The net effect of ASA in the prevention of CVD and cancer is a secondary outcome measure. Finally, Vitamin D will be evaluated because Asian populations are considered to be deficient in Vitamin D, and several guidelines recommend its use despite the lack of an RCT showing clinical benefit in these populations.7., 8. In this paper, our focus is on the comparison of the Polycap versus its placebo.

### Study population and eligibility

Eligibility criteria was based on absence of CVD, age, and the non-laboratory based INTERHEART risk score (IHRS), which is a validated tool for estimating CVD risk in multiple populations, without the need for laboratory-based measures (eg, cholesterol).9., 10. We included participants who were at least at intermediate risk of developing CVD based on their age and IHRS. Community-dwelling participants were recruited from primary care clinics, specialty clinics, or community outreach programs. Detailed information on study inclusion and exclusion criteria are summarized in Table I.

**Table I:** Inclusion and exclusion criteria of TIPS-3

Inclusion criteria:	1. Men aged ≥50 years and women aged ≥55 years with an INTERHEART risk score ≥ 10, or men and women aged ≥65 years with an INTERHEART risk score of ≥5.⁎ 2. Provision of informed consent
Exclusion criteria:	1. Participants with a clear clinical indication, contraindication, preference for or intolerance to statin, beta blocker (eg, bradycardia), ACE inhibitor, diuretic, aspirin or clopidogrel in the judgment of the physician. 2. Regular use of vitamin D at doses higher than 400 IU per day. 3. Hypercalcemia, hyperparathyroidism, osteomalacia or other contraindication or indication for vitamin D therapy. 4. Peptic ulcer disease, frequent dyspepsia or bleeding. 5. Expected long term use of anticoagulants 6. Known vascular disease. (eg, Stroke, TIA, Angina, MI, ACS, PVD including claudication and amputation). 7. Mean systolic BP below 120 mm Hg at run-in. 8. Symptomatic hypotension (eg, dizziness with SBP <110 mm Hg systolic) during the run-in phase. 9. Chronic liver disease or abnormal liver function, i.e. ALT or AST >3 × ULN. 10. Inflammatory muscle disease (such as dermatomyositis or polymyositis) or creatine kinase (CK) >3 × ULN. 11. Severe renal impairment (serum creatinine >264 μmol/L). 12. History of malignancy affecting any organ system, except basal cell carcinoma of the skin, within the previous 5 years. 13. Other serious condition(s) likely to interfere with study participation or with the ability to complete the trial. 14. Concurrent use of any experimental pharmacological agent. 15. Inability to attend follow-up as required by the protocol for at least 5 years.

⁎ The original inclusion criteria for the study was men aged ≥55 years and women aged ≥60 years with an INTERHEART risk score ≥10. This was revised in February 2015 to include individuals at lower ages, as well as higher age groups with a lower INTERHEART risk score. This would still reflect an intermediate risk population (i.e. annual event rate >1%/year) since age is the strongest risk factor for CVD.

### Primary, secondary, and additional pre-specified outcomes

#### Polycap versus placebo

The primary study outcome for this comparison is the composite of CV events, which includes major CVD (ie, CV death, non-fatal stroke, non-fatal MI), plus heart failure, resuscitated cardiac arrest, or arterial revascularization. Secondary outcomes are (1) major CVD and (2) the composite of major CVD, heart failure, resuscitated cardiac arrest, arterial revascularization, or angina with evidence of ischemia.

#### Aspirin versus placebo

The primary outcome of this comparison is the composite of major CVD (ie, CV death, non-fatal stroke, non-fatal MI). The secondary outcome is the composite of major CVD and cancer.

#### Vitamin D versus placebo

The primary outcome of this comparison is fractures. The secondary outcome is the composite of CV events (as described in Section 2.3.1), fractures, cancers, and falls.

#### Combined effects of the Polycap and aspirin

The primary outcome of this comparison is major CVD (CV death, non-fatal MI or non-fatal stroke), heart failure, resuscitated cardiac arrest, or arterial revascularization. Secondary outcomes are (i) major CVD and (ii) the composite of major CVD (CV death, non-fatal stroke, non-fatal myocardial infarction [MI]), heart failure, resuscitated cardiac arrest, arterial revascularization, or angina with evidence of ischemia.

#### Additional outcomes

Additional pre-specified outcomes include all-cause mortality, incident and recurrent CV events, visual acuity, age-related macular degeneration, cognitive function, adverse events (including bleeding), and economic analysis related outcomes.

TIPS-3 was started prior to the results of the HOPE-3 trial, which was published in 2016, and showed a benefit of statin therapy over placebo in individuals at intermediate CVD risk. After the results of HOPE-3 were published, the TIPS-3 steering committee decided to continue the current study design for several reasons. First, since HOPE-3 was the first long-term clinical trial to demonstrate this effect in an intermediate CVD risk primary prevention population, it was felt that confirmation of these findings were needed prior to widespread adoption of such a strategy, which TIPS-3 can provide. Second, indications for statin use continue to vary between clinical guidelines in different countries, and even generic statins are relatively unaffordable in many LICs and MICs, and so their use even in secondary prevention is low.11., 12. Third, investigators have the option of discontinuing the Polycap and starting open label medications if a participant meets an indication for statin therapy based on local practice.

### Sample size and data analysis

Reductions in cholesterol and blood pressure levels observed with the Polycap in prior studies suggest that a reduction in CV events of at least 35% is feasible, and likely necessary to be accepted in clinical practice (as lesser benefits can be achieved by using one BP lowering drug and a statin given separately). The study was originally designed to enroll 5000 participants over two-years, with a further 4 years of follow-up resulting in a mean follow-up of 5 years. Assuming a CV event rate of 1.0%/year in the placebo group, the study would have over 80% power to detect a 35% RRR in CV events with the Polycap compared to placebo, and over 90% to detect a 40% RRR. Ultimately recruitment required 5 years, and was skewed towards a higher enrollment in the final years. To compensate for this, a total of 5713 participants were enrolled in the study, and the anticipated completion of study follow-up will potentially be extended to a mean follow-up of up to approximately 4.25 years. The observed overall annual CV event rate in the study was 1.1% at the end of the recruitment phase. Based on this revised data, with extension of the study, it will maintain at least 80% power to detect a 35% RRR reduction in CV events, and over 90% power to detect a 40% RRR, comparing the Polycap to placebo. Further calculations outlining the statistical power of the study are available in the supplementary appendix of this paper.

The primary analysis for each treatment group will be based on the principle of intention to treat. For each comparison, survival curves for the primary and secondary outcomes will be generated using the Kaplan–Meier procedure. The primary analysis will be the time to a confirmed primary outcome event using the Cox proportional hazards model. Comparisons will be presented using hazard ratios with 95% confidence intervals, and a two-sided p-value of <0.05 will be considered statistically significant. Possible interactions between treatments will be tested by the inclusion of interaction terms in the model. Although interactions between the study medications are not anticipated, in the unlikely event of a significant interaction, treatment effects will be reported separately for each strata defined by the interacting treatment. Consistency of treatment effects on each primary outcome will be explored in a few predefined subgroups, including by thirds of pre-treatment LDL-cholesterol and blood pressure levels, thirds of the IHRS, gender, ethnicity and the presence or absence of dysglycemia (ie, diabetes or impaired fasting glucose). Whether treatments effects vary by subgroups will be analyzed using tests for interactions in the Cox regression model.

### Study procedures

Following consent, eligible participants underwent a 3 to 4 week run-in phase, during which time they received low dose Polycap (consisting of atenolol 50 mg, ramipril 5 mg, HCTZ 12.5 mg and simvastatin 40 mg) and low dose aspirin daily. Participants who tolerated the study medications and did not meet run-in exclusion criteria were randomized to each of the study medications or their matching placebos. Allocation concealment was maintained by using a central randomization process that was stratified by center with fixed randomization blocks (of 8 participants). Follow up visits occur at 6 weeks, 3 months, 6 months, 9 months, 1 year, then at 6-month intervals until the end of the study. Blood pressure readings were collected prior to run-in, at randomization and during the follow up. Fasting lipids were collected prior to run-in and during follow up. As part of our pre-specified study outcomes, tools for measuring visual acuity, cognitive function, and quality of life were performed at baseline, and will be repeated during follow-up.

### Study organization

The TIPS-3 study is being conducted at 86 centers in 9 countries. The study is funded through grants by the Wellcome Trust, Canadian Institutes for Health Research, Cadila pharmaceuticals, the Population Health Research Institute (PHRI), Heart and Stroke Foundation of Ontario, Philippines Council for Health Research and Development, Secretaria de Salud del Departamento de Santander (Colombia) and St. John's Research Institute (India). Ethics approvals were obtained at all participating centers, and regulatory approvals for conducting the trial and importation of the study drugs for the trial were obtained for each country. Written informed consent has been provided by all participants. Trial oversight occurs by an international steering committee comprised of the study's principal investigator and several co-investigators (see supplementary appendix). The central coordinating center of the study is the PHRI, Hamilton Health Sciences and McMaster University, in Hamilton, Ontario, Canada. Data are stored at the coordinating center using a secure electronic database, called iDataFax. Simple data collection forms are being used to collect baseline and follow-up data, which are sent to the central coordinating center via fax or entered at the site using the iDatafax software. To ensure data quality, automated checks have been developed within the software itself, and additional checks are performed at the coordinating center. An independent data monitoring and safety board, assisted by a senior biostatistician who is independent of the everyday conduct of the trial, oversees the safety of each treatment and study conduct at six-month periods. In addition, three formal interim efficacy analyses are planned based on the number of primary outcome events that are expected to have accrued (see supplementary appendix for details of statistical guidelines and monitoring boundaries). Supporting documentation (eg, hospitalization records, diagnostic tests, and procedural notes) is requested for all primary outcome events, which then undergo adjudication by a committee that is blinded to the treatment assignments and using pre-specified criteria.

## Results

### Study enrollment

Of 7701 screened participants, 7539 were eligible for run-in. Of these 1826 (24.2%) were not eligible for randomization, resulting in 5713 participants being randomized to the study. The most common reason for not undergoing randomization was participant decision (20.6%), followed by <80% adherence to the Polycap (11.2%) or aspirin (10.9%). Only 4.7% of participants were ineligible due to syncope, dizziness or a SBP <110 mmHg. Only 1.4% were ineligible due to elevated blood tests meeting exclusion criteria; and 1% were ineligible due to peptic ulcer disease, dyspepsia or gastrointestinal bleeding.

### Baseline characteristics

Key baseline characteristics of the study population are summarized in Table II. Most participants were recruited in India (47.9%) followed by the Philippines (29.3%). The mean age of the study population was 63.9 years, and 53.0% were female. A history of hypertension was self-reported in 77.6% of participants, and diabetes was reported in 32.2%. 1961 (34.3%) participants had a fasting glucose ≥6.1 mmol/L).

**Table II:** Baseline characteristics of the 5713 participants enrolled in TIPS-3

Variable	
Mean age, years (SD)	63.9 (6.6)
Female, N (%)	3026 (53.0%)
Country of recruitment, N (%)	
India	2739 (47.9)⁎
Philippines	1676 (29.3)
Colombia	489 (8.6)
Bangladesh	295 (5.2)
Canada	131 (2.3)
Malaysia	119 (2.1)
Indonesia	118 (2.1)
Tunisia	107 (1.9)
Tanzania	39 (0.7)⁎
Risk factors:	
Self-reported hypertension, N (%)	4436 (77.6)
Self-reported diabetes, N (%)	1841 (32.2)
Fasting glucose ≥6.1 mmol/L (%)	1961 (34.3)
Current smoker, N (%)	512 (9)
Mean INTERHEART Risk Score	16.8 (4.6)
Physiologic parameters:	
Mean heart rate, beats per minute (SD)	77.0 (10.6)
Mean systolic blood pressure, mmHg (SD)	144.5(16.8)
Mean diastolic blood pressure, mmHg (SD)	83.9(9.7)
Mean total cholesterol, mmol/L (SD)	5.1 (1.2)
Mean low density lipoprotein, mmol/L (SD)	3.1 (1.1)
Mean high density lipoprotein, mmol/L (SD)	1.2 (0.3)
Mean triglycerides, mmol/L (SD)	1.6 (0.8)
Mean fasting plasma glucose, mmol/L (SD)	6.3 (2.5)
Mean Creatinine, mmol/L (SD)	81.5 (23.3)
Mean BMI, kg/m^2^ (SD)	25.8 (4.8)
Mean waist-to-hip ratio (SD)	
Males	0.96 (0.06)
Females	0.91 (0.07)

⁎ After randomization, all patients from Tanzania (n = 39) and a small number in India (N = 18) were withdrawn because of regulatory barriers resulting in site closures. Participants at these sites were censored at the time of the site closure. Currently 5656 participants are actively in follow-up.

## Regulatory factors, study initiation and study enrollment

### Time required to achieve regulatory approval in each country

Approval to conduct the study was granted in 10 countries, of which 9 enrolled participants (approval was also granted by the Food and Drug Administration, United States of America, although the study was not operationalized in this country). Approval times to start the study varied substantially, with Tunisia and Colombia granting approval in <3 months; India, Philippines, and Malaysia taking 3 to 6 months to approve the study; Canada and Bangladesh requiring approximately 9 months for approval; and Indonesia and Tanzania requiring approximately 1 year for approval. In addition, submissions were withdrawn in 3 countries (Brazil, China, and Argentina) after facing multiple challenges to approving the study despite extensive efforts over a 2-year period.

### Regulatory changes in India and enrollment trends in TIPS-3

Between 2013 and 2014, several new regulatory requirements in India were created that directly impacted the conduct of ongoing clinical trials. These included a requirement for compensation for trial related injury or death, clinical trial inspections, audiovisual (AV) recording of the informed consent process, and limitations on the number of clinical trials that could be performed by an investigator.13., 14. In 2013, 22 sites within India were actively recruiting participants in TIPS-3. These regulatory changes contributed to the closure of 5 study sites. While most sites continued, the challenging regulatory environment was commonly cited by investigators as a reason for higher operational costs, greater complexity of recruitment, and a substantial decline in recruitment rates. These changes also negatively impacted our ability to identify additional sites that were willing to participate in the study. Several of these regulatory requirements were subsequently amended (i.e. clarification of compensation for trial related injury, relaxing AV consent requirments) between 2015 and 2016.

**Figure 2:**
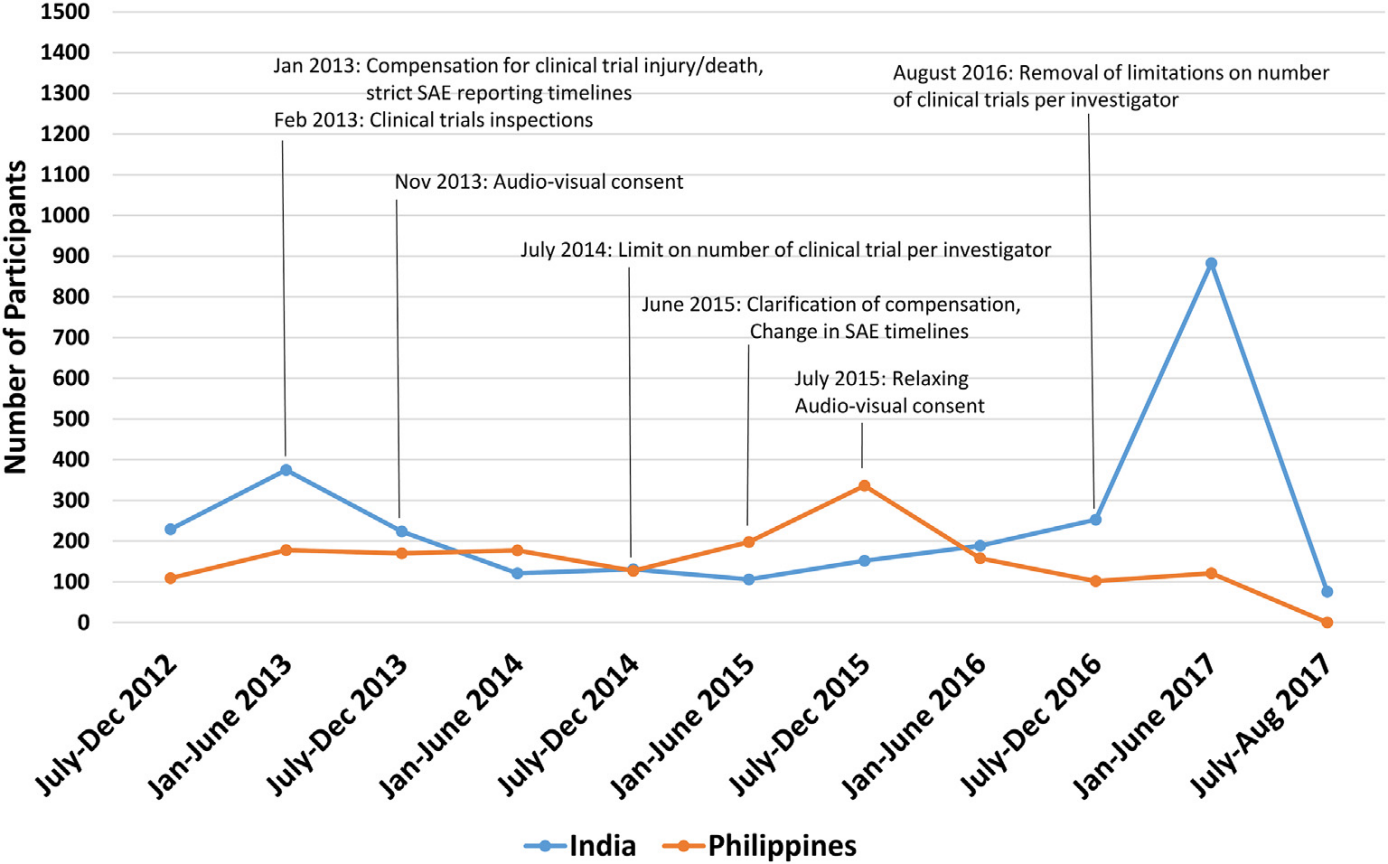
Number of participants randomized in six-month intervals within India and the Philippines, and in relation to major clinical trial regulatory changes that occurred within India.13., 14. Several regulatory changes that started in 2013 contributed to a reduction in randomization in India until approximately June 2015. The large increase in recruitment that occurred in 2017 was also partly the result of several new sites joining TIPS-3.

A summary of study recruitment in India at 6-month intervals is provided in Figure 2. For comparison, a summary of recruitment in the Philippines (the second highest recruiting country in our study) is also provided. After the introduction of regulatory changes in early 2013, a substantial and prolonged decrease in recruitment occurred until late 2015, when many of the introduced changes were relaxed. During this time, from a peak 6 month enrollment of 375 participants (occurring from January to June 2013), enrollment declined by 72% to a trough of 106 participants (January to June 2015). Between 2013 and 2016, enrollment in the Philippines also declined but to a lesser extent (maximum decline of 32% per 6-month period), and recovered at a faster rate when compared with India.

## Discussion

TIPS-3 will be the first large clinical trial to examine whether FDC therapy (using the Polycap) targeting aggressive blood pressure and cholesterol reduction is effective in the primary prevention of CVD in individuals at increased risk. This study will address key knowledge gaps that currently exist and limit the use of FDC therapy in the primary prevention of CVD. First, estimates of the benefits of FDC therapies are largely extrapolated from their impacts on blood pressure and cholesterol levels. However, determining their actual effects on CVD risk requires their examination in long-term clinical outcome trials, such as TIPS-3.^2^ Second, TIPS-3 will determine the tolerability of FDC therapy in a broad range of participants at increased CVD risk, across several populations in LICs and MICs where its use is likely to be most applicable. Third, we will examine whether effects differ by key risk factor levels (eg, blood pressure, lipids, overall CVD risk) to better inform its application in primary CVD prevention.

Control of common risk factors for CVD is suboptimal in many regions of the world, particularly in LICs and MICs.^15^ If a 35% to 40% reduction in CVD outcomes can be achieved with the Polycap, global adoption could potentially avert up to 10–13 million cases of CVD per year. Importantly, implementation of a FDC strategy as part of CVD prevention can have a significant impact on how CVD is managed across a wide range of health resource settings. In high-income countries, FDC therapy could address common barriers to medication non-adherence, such as the complexity of medication regimens, as well as cost.^16^ Medication access and affordability are more wide-spread barriers to CVD prevention in MICs and LICs, and the broad adoption of low-cost, FDC therapy by healthcare payers and providers in these regions could be a central strategy to CVD control at the population level.17., 18., 19.

We have also provided a brief example of how changes in national regulations can adversely impact enrollment in a multi-national clinical trial. The highest priority of clinical trial regulation is ensuring that research participants are treated safely and ethically, while balancing the need to conduct ethical research in a productive manner. We identified two instances where regulatory factors significantly and adversely impacted the ability to conduct our clinical trial. The first was during initial approval, where we faced prolonged regulatory processes, resulting in significant delays to commencing the study in multiple countries. Furthermore, three countries did not provide approvals despite prolonged efforts over a 2-year period and repeated responses to questions from regulators. The large variation in time required to obtain regulatory approval between countries reflect the substantial differences in processes that currently exist, and highlights the need to better streamline current practices in several countries. Furthermore, this may have also reflected discomfort related to the concept of using a polypill (with 4 components) on the part of some regulators, despite the fact that all the components of the Polycap are safe, effective, and widely used in clinical practice; and that the Polycap was found to be well tolerated in two prior clinical studies.20., 21. The second instance occurred during the implementation of new regulatory guidelines in India that substantially increased the efforts and risk undertaken by investigators to participate in clinical research; and the complexity and costs of conducting the trial. Although we acknowledge that other factors impacted recruitment rates in India (eg, the addition of study centers, the relatively modest funding), trends in enrollment during the period in which the most restrictive regulatory requirements came into force strongly suggest that such regulatory policies dramatically impacted study conduct. Our data are also consistent with other analyses showing that the number of registered phase II or III clinical trials in India had decreased by >70% between 2013 and 2016.^22^ Importantly, many of these policies were amended to a more pragmatic set of policies (without compromising ethical conduct or participant safety), but this process required over 2 years. These data show the profound impact that regulatory changes can have on the conduct of scientific research.

In addition to the above challenges, substantial regulatory barriers to the importation of study drugs have occurred in several countries, which has resulted in delays and interruptions in patients taking them. In some instances, this has led to worsening adherence of participants to the study medications, and additional efforts on the parts of the participants and local investigators (eg, added study visits), national leaders (eg, clearing drugs through customs, obtaining repeated approvals for importation for each batch of study drugs), staff at the coordinating center (eg, reallocating drugs to minimize the impact of a lack of availability), and the drug distribution team at Cadila (who have to obtain approval from the Drugs Controller General of India for each drug shipment outside India). These challenges also led to prolonged delays in receiving study drugs in Tanzania, and contributed to the study being stopped early in this country due to administrative delays. In future analyses of TIPS-3, characterizing the potential impact of temporary study drug discontinuation on clinical outcomes may be considered. As clinical trials research continues to expand to more regions of the world, there is a need to have greater collaboration between the scientific community and regulators (especially in LICs and MICs) in order to develop balanced regulatory and importation processes that do not compromise ethical integrity or participant safety, but are also designed to avoid unnecessary and onerous barriers to the conduct of scientific studies.

## Conclusions

Results of TIPS-3 will be key to determining the appropriateness of FDC therapy as a strategy in the global prevention of CVD. If the study demonstrates that the Polycap reduces the risk of CVD by at least 35%, then the polypill will likely gain acceptance as a cost effective and convenient approach for CVD prevention.

## Conflicts of Interest and Disclosures

None of the authors have any relevant conflicts of interest related to the study. SY and PP have received research grants from Cadila pharmaceuticals. SY has received honoraria from several companies (Bayer, Boehringer Ingelheim, Astra Zeneca, Ferrer) for separate activities unrelated to any of the treatments being evaluated in the trial. JB has received honoraria from Bayer. PLJ has received honoraria from Ferrer and Scandinavia pharmaceutical laboratories.

## Funding of Study

TIPS-3 is funded through unrestricted grants by the Wellcome Trust, Canadian Institutes for Health Research (#259128), Cadila pharmaceuticals, the Population Health Research Institute (PHRI), Heart and Stroke Foundation of Ontario (#000448), Philippines Council for Health Research and Development, Secretaria de Salud del Departamento de Santander (Colombia) and St. John's Research Institute (India).

## Author Contributions

All authors contributed significantly to the work. Drs. PJ, PP, TK and SY contributed to the concepts of the paper. Drs. PJ, TK and SY contributed to data analysis or interpretation. Drs. PJ, PP, AD, JB, DX, PLJ, KY, ST, AS, HG, KY, PCL, KT, and SY contributed to drafting the manuscript, or revising it critically for intellectual content. All authors provided final approval of the manuscript.

## Appendix A Supplementary data

Supplementary material Image 1
