# Supplementary material for: The International Polycap Study-3 (TIPS-3): Design, baseline characteristics and challenges in conduct
Source: Am Heart J. 2018 Dec;206:72–9. doi: 10.1016/j.ahj.2018.07.012 (PMC6299262; doi:10.1016/j.ahj.2018.07.012)
Supplement: Supplementary file 1 — Supplementary material [file mmc1.pdf]

Supplementary Appendix: The International Polycap Study (TIPS-3): design, baseline characteristics and challenges in conduct

Sample size and study power calculations to determine the effect of the Polycap compared to placebo:

Originally, the trial was planned to randomize 5000 study participants and follow them for a mean of 5 years, which would have >80% power for a 35% reduction, >90% power for a 40% reduction in composite CV events at 5 years (supplemental table 1). The sample size calculation was based on an estimated yearly event rate of 1% for major CVD events in the placebo group; non-adherence rates in the intervention arm of 8% in the first year, an additional 3% in the second year, and an additional 2% per year for subsequent years for each active therapy (17% at 5 years); drop-in rates of 2% per year (10% over 5 year; and rates of lost to follow-up of < 2 % at 5 years. We have set the two-sided alpha at 0.05.

SUPPLEMENTARY TABLE 1: ORIGINAL SAMPLE SIZE CALCULATIOMS ASSUMING TWO YEARS OF RECRUITMENT, PLUS FOUR YEARS OF FOLLOW-UP (MEAN FOLLOW-UP OF 5 YEARS) WITH AN 18% DROPOUT RATE, AND A 10% DROP IN RATE.

		**Power (%)**	
**Risk Reduction (%)**	**Control Event Rate (CV death, MI, stroke, heart failure, revascularization, resuscitated cardiac arrest)**	**80**	**90**
35	1.0%/year	4224	5654
40	1.0%/year	3132	4194
50	1.0%/year	1876	2512

The trial has enrolled 5713 participants, and assumptions were revised based on the trial experiences to date. The following modifications were made to estimate study power: a total trial period of approximately 8 years, control event rate of 1.2% to 1.3% per year (assuming a treatment effect exists with an overall event rate of 1.1% observed so far), drop-out rate of 29% at 8 years, drop in rate of 8% at 8 years (instead of 17% and 10% respectively), and loss to follow up of 2% at 8 years. Based on these modifications, the study will still have >80% power for a 35% reduction, >90% power for a 40% reduction in composite CV events (supplemental table 2).

SUPPLEMENTARY TABLE 2: REVISED SAMPLE SIZE CALCULATIONS BASED ON TRIAL PERFORMANCE AT THE END OF THE ENROLLMENT PHASE:

Risk Reduction (%)	Control Event Rate (CV death, MI, stroke, heart failure, revascularization, resuscitated cardiac arrest)	Power (%)
35	1.2%/year	83.7
	1.3%/year	86.6
40	1.2%/year	92.5
	1.3%/year	94.4

Statistical considerations for interim analyses:

An independent Data Safety and Monitoring Board (DSMB) will review all data on safety and efficacy at regular intervals (every 6 months). Three formal interim analyses are planned, equally spaced with respect to accumulating number of events. If the interim analyses show clear benefit, the DSMB may recommend early trial termination. A conservative stopping rule approach will be recommended and will be formalized in conjunction with the DSMB at their first meeting. A suggested guideline for early termination for efficacy is a reduction of 4 standard deviations (corresponding to a p value of 0.00006) in the primary outcomes at the first and second looks, or

3 standard deviations (corresponding to a p value of 0.0027) at the third look. Conversely, a 3 standard deviation excess in the primary study outcomes with treatment at the first two looks and 2 standard deviations excess at the third look would be considered evidence for harm. The monitoring boundaries should remain crossed at 2 successive analyses at least 3 months apart to ensure that temporary variations in the effect sizes do not lead to early termination. Given these extremely conservative monitoring boundaries and the limited number of formal interim analyses, the type I error rate adjustment for the final analysis will be negligible (<0.00001). If the study is discontinued for one component, the trial will continue evaluating the other components.

TIPS-3 Steering Committee Members:

Name	Country
Bosch, Jackie	Canada
Dans, Antonio	Philippines
Gamra, Habib	Tunisia
Gogia, Mona****	India
Maseeh, Arun (left steering committee during the study) ****	India
Joseph, Philip	Canada
Lopez-Jaramillo, Patricio	Colombia
Mukherjee, Shirshendu***	India
Seabrook, Richard***	United Kingdom
Tay, Diana***	United Kingdom
Pais, Prem**	India
Santoso, Anwar	Indonesia
Talukder, Shamim	Bangladesh
Teo, Koon**	Canada
Xavier, Denis	India
Yeates, Karen	Tanzania
Yusoff, Khalid	Malaysia
Yusuf, Salim*	Canada

*Principal Investigator, **Co-principal Investigator, ***Wellcome Trust, ****Cadila Pharmaceuticals.

TIPS-3 Site Principal and Co-Investigators:

Country	Name	Center	Role
Bangladesh	Shamim Hayder Talukder	Eminence (401-409)	PI
Bangladesh	Shahin Akter	Eminence (401-405)	Co-PI
Bangladesh	Rubel Rahman Babu	Eminence (406)	Co-PI
Bangladesh	MD Rasheduzzaman	Eminence (407)	C0-PI
Bangladesh	Moushumy Popy	Eminence (408)	Co-PI
Bangladesh	Tabassum Tamanna	Eminence (409)	Co-PI
Canada	Carolyn Taylor	St. Paul's Hospital	PI
Canada	Scott Lear	St. Paul's Hospital	Co-PI
Canada	Eva Lonn	Hamilton General Hospital	PI
Canada	Gilles Dagenais	Institut Universitaire De Cardiologist	PI
Canada	Paul Poirier	Institut Universitaire De Cardiologist	Co-PI
Canada	Pravinsagar Mehta	Dr. Pravinsagar Mehta - Laxmi Centre	PI
Canada	Preston Zuliani	Dr. Preston Zuliani	PI
Canada	Michael Walsh	St. Joseph's Healthcare	PI
Canada	Nader Khalidi	St. Joseph's Healthcare	Co-PI
Colombia	Paul Anthony Camacho Lopez	FOSCAL	PI
Colombia	Gustavo Adolfo Parra	FOSCAL	Co-PI
Colombia	Andres Alirio Serrano Barco	FOSCAL	Co-PI
Colombia	Leonardo Fabio Forero-Naranjo	FOSCAL	Co-PI
Colombia	Jose Accini	Centro Científico Asistencial	PI
Colombia	Melissa Accini Valencia	Centro Científico Asistencial	Co-PI
Colombia	Gregorio Sanchez	Fundacion Cardiomed, Universidad del Quindio	PI
Colombia	Juan Pablo Yepez	Fundacion Cardiomed, Universidad del Quindio	Co-PI
Colombia	Dora Ines Molina de Salazar	IPS Internistas de Caldas, Universidad de Caldas	PI
Colombia	Luisa Jhoanna Suarez Correa	IPS Internistas de Caldas, Universidad de Caldas	Co-PI
Colombia	German Carmilo Graldo Gonzalez	IPS Internistas de Caldas, Universidad de Caldas	Co-PI
Colombia	Carlos Eduardo Rivera Molano	IPS Internistas de Caldas, Universidad de Caldas	Co-PI
Colombia	Catalina Lopez Ceballos	Centro de Investigacion Clinica CARDIOMED-Pereira	Co-PI

Country	Name	Center	Role
Colombia	Luis Garcia	Centro de Investigacion Clinica CARDIOMED-Pereira	PI
Colombia	Carlos Montoya	Centro de Investigacion Clinica CARDIOMED-Pereira	Co-PI
India	Hitesh Patel	Saviour Multispeciality Hospital	PI
India	Bhavik Prajapati	Saviour Multispeciality Hospital	Co-PI
India	Ganapathi Bantwal	St. John's Medical College	PI
India	Vageesh Ayyar	St. John's Medical College	Co-PI
India	Belinda George	St. John's Medical College	Co-PI
India	Mahmood Saba Fatima	St. John's Medical College	Co-PI
India	Mathew Vivek	St. John's Medical College	Co-PI
India	Sindhu Joshi	Mahavir Hospital & Research Centre	PI
India	D Krishnamurthy	Mahavir Hospital & Research Centre	Co-PI
India	Viswanathan Mohan	Madras Diabetes Research Foundation	PI
India	Kasthuri Selvam	Madras Diabetes Research Foundation	Co-PI
India	BK Gupta	S.P. Medical College & A.G. Hospitals	PI
India	DK Agarwal	S.P. Medical College & A.G. Hospitals	Co-PI
India	BL Ranka	S.P. Medical College & A.G. Hospitals	Co-PI
India	Jigyasa Gupta	S.P. Medical College & A.G. Hospitals	Co-PI
India	Mukesh Kumar Sarna	Fortis Escorts Hospital	PI
India	Rajeev Gupta	Fortis Escorts Hospital	Co-PI
India	Sandeep Kumar Gupta	M.V. Hospital and Research Centre	PI
India	Danesh Chandra Pandey	M.V. Hospital and Research Centre	Co-PI
India	Ashok Kumar Mishra	M.V. Hospital and Research Centre	Co-PI
India	Urmil Shah	Care Institute of Medical Sciences	PI
India	Anish Chandarana	Care Institute of Medical Sciences	Co-PI
India	Parthasarthy Iyengar	Care Institute of Medical Sciences	Co-PI
India	Keyur Parikh	Care Institute of Medical Sciences	Co-PI
India	Milan Chag	Care Institute of Medical Sciences	Co-PI
India	Hemang Baxi	Care Institute of Medical Sciences	Co-PI
India	Satya Gupta	Care Institute of Medical Sciences	Co-PI
India	Ajay Naik	Care Institute of Medical Sciences	Co-PI
India	Jyoti Bhatia	Care Institute of Medical Sciences	Co-PI
India	Guarav Chhaya	Sanjivani Superspeciality Hospital	PI
India	Vinay Bhomia	Sanjivani Superspeciality Hospital	Co-PI
India	Naveen Reddy	Mediciti Hospitals	PI
India	Sheela Thorat	Mediciti Hospitals	Co-PI
India	Kamlesh Fatania	Rathi Hospital	PI
India	Gaurav Rathi	Rathi Hospital	Co-PI

Country	Name	Center	Role
India	Muthu Ramu	Dr. Mohan's Diabetes Specialities centre	PI
India	Selvakumar Jeyavel	Dr. Mohan's Diabetes Specialities centre	Co-PI
India	Sudhir Kumar Bhatnagar	Abhinav Hospital	PI
India	Anil Jawahjrani	Abhinav Hospital	Co-PI
India	Neeta Deshpande	Belgaum Diabetes Centre	PI
India	Aman Syed	Belgaum Diabetes Centre	Co-PI
India	Kamal Sharma	Dr. Kamal Sharma Cardiologist Clinic	PI
India	Neha Sharma	Dr. Kamal Sharma Cardiologist Clinic	Co-PI
India	Ketan Mehta	Health Harmony	PI
India	Arvind Ghongane	Health Harmony	Co-PI
India	Devavrat Shahasane	Health Harmony	Co-PI
India	Bandari Srinivasulu	Aditya Nursing Home	PI
India	Byalla Janaki Srinivas	Aditya Nursing Home	Co-PI
India	Bosco Balraj	Trichy Diabetes Centre	PI
India	Balamurugan Mallingar	Trichy Diabetes Centre	Co-PI
India	Ravindranath Venkatesan	Trichy Diabetes Centre	Co-PI
India	J Narendra	Nanjappa Life Care	PI
India	B.H. Lokesh	Nanjappa Life Care	Co-PI
India	Yerrathota Balaji	Sree Balaji Nursing Home	PI
India	Yerrathota Bhavishya	Sree Balaji Nursing Home	Co-PI
India	Damodara Rao Kodem	Queen's NRI Hospital	PI
India	Venkata Venu Gopal Anumanchipalli	Queen's NRI Hospital	Co-PI
India	Bhaskara Rao Malipeddi	Queen's NRI Hospital	Co-PI
India	Rajeev Gupta	Eternal Heart Care Centre	PI
India	Raghubir Singh Khedar	Eternal Heart Care Centre	Co-PI
India	Jugal Bihari Gupta	Eternal Heart Care Centre	Co-PI
India	Geeti Arora	Deep Heart Centre	PI
India	Rakesh Aggarwal	Deep Heart Centre	Co-PI
India	Abraham Oomman	Ramana Maharishi Rangammal Hospital	PI
India	Fazel Mohammed Saleem	Ramana Maharishi Rangammal Hospital	Co-PI
India	Kannan Balamurali	Ramana Maharishi Rangammal Hospital	Co-PI
India	Sudha Vidyasagar	Manipal Centre for Clinical Research	PI
India	Muralidhar Varma	Manipal Centre for Clinical Research	Co-PI
India	Kavitha Saravu	Manipal Centre for Clinical Research	Co-PI
India	K.M. Srinath	JSS Medical College Hospital	PI

Country	Name	Center	Role
India	B. Madhu	JSS Medical College Hospital	Co-PI
India	Vellala Elumalai Dhandapani	SRM Medical College	PI
India	Melvin George	SRM Medical College	Co-PI
India	Subramaniyan Kumarasamy	SRM Medical College	Co-PI
India	Shrikant Kulkarni	Noble Hospital	PI
India	Namdev Jagtap	Noble Hospital	Co-PI
India	Mukund Penurkar	Sanjeevan Hospital	PI
India	P.T. Chandrahas	Saptagiri Institute of Medical Sciences	PI
India	Apurva Patel	SAL Hospital	PI
India	Anil Jain	SAL Hospital	Co-PI
India	Nirmal Parmar	SAL Hospital	Co-PI
India	Dushyant Balat	Apollo Hospitals International Limited	PI
India	Sameer Dani	Apollo Hospitals International Limited	Co-PI
India	Piyush Desai	Nirmal Hospital Pvt. Ltd.	PI
India	Mehul Bhavsar	Nirmal Hospital Pvt. Ltd.	Co-PI
India	Vikrant Vijan	Vijan Cardiac & Critical Care Centre	PI
India	Ganesh Wadgoankar	Vijan Cardiac & Critical Care Centre	Co-PI
India	Pravin Dinkar Supe	Supe Heart & Diabetes Hospital	PI
India	Anupama Pravin Supe	Supe Heart & Diabetes Hospital	Co-PI
India	Varade Deepak	Asian Institute of Medical Sciences	PI
India	Sunder Thiruginana Sambandan	Apollo Research & Innovations	PI
India	Kannaiyan Ramamurthee	Apollo Research & Innovations	Co-PI
India	Abraham Dr. Oomman	Apollo Research & Innovations	Co-PI
India	Gursaran Sidhu	Sidhu Hospital	PI
India	Shivali Anand	Sidhu Hospital	Co-PI
India	Abhitej Sidhu	Sidhu Hospital	Co-PI
India	Vineet Shukla	KRM Hospital	PI
India	Nalini Mishra	KRM Hospital	Co-PI
India	Naveen Jamwal	KRM Hospital	Co-PI
India	Sanjeev Mohan	KRM Hospital	Co-PI
India	Sandeep Gupta	KRM Hospital	Co-PI
Indonesia	Anwar Santoso	Harapan Kita Hospital	PI
Indonesia	Ade Meidian Ambari	Harapan Kita Hospital	Co-PI
Indonesia	Suko Adiarto	Harapan Kita Hospital	Co-PI
Indonesia	Basuni Radi	Harapan Kita Hospital	Co-PI
Indonesia	Bambang Widyantoro	Harapan Kita Hospital	Co-PI

Country	Name	Center	Role
Malaysia	Khalid Yusoff	Universiti Teknologi Mara (UiTM) and UCSI University	PI
Malaysia	Nafiza Mat Nasir	Universiti Teknologi Mara (UiTM)	Co-PI
Malaysia	Farnaza Ariffin	Universiti Teknologi Mara (UiTM)	Co-PI
Malaysia	Khairul Shafiq Ibrahim	Universiti Teknologi Mara (UiTM)	Co-PI
Malaysia	Ambigga S. Krishnapillai	Universiti Pertahanan Malaysia (UPNM)	Co-PI
Malaysia	NG Kien Keat	Universiti Pertahanan Malaysia (UPNM)	Co-PI
Malaysia	Johan Rizwal Ismail	Universiti Teknologi Mara (UiTM) - Kasim	PI
Malaysia	Nicholas Chua Yul Chye	Universiti Teknologi Mara (UiTM) - Kasim	Co-PI
Malaysia	Raja Ezman Fardiz bin Raja Shariff	Universiti Teknologi Mara (UiTM) - Kasim	Co-PI
Malaysia	Mohd Aznan	International Islamic University Malaysia	PI
Malaysia	Fa'iza Abdullah	International Islamic University Malaysia	Co-PI
Malaysia	Samsul Draman	International Islamic University Malaysia	Co-PI
Malaysia	Nurjasmine Jamani	International Islamic University Malaysia	Co-PI
Philippines	Homer Co	Philippines General Hospital	PI
Philippines	Lia Aileen Palileo	Philippines General Hospital	Co-PI
Philippines	Lia Aileen Palileo	Philippines General Hospital	Co-PI
Philippines	Marc Evans Abat	Philippines General Hospital	Co-PI
Philippines	Michael Joseph Agbayani	Philippines General Hospital	Co-PI
Philippines	Antonio Miguel Dans	Philippines General Hospital	Co-PI
Philippines	Martha Jane Pauline Umali	Philippines General Hospital	Co-PI
Philippines	Aldrin Loyola	Philippines General Hospital	Co-PI
Philippines	Aldrin Loyola	Philippines General Hospital	Co-PI
Philippines	Dante Morales	Manila Doctors Hospital	PI
Philippines	Daniel Francisco Morales	Manila Doctors Hospital	Co-PI
Philippines	Ronald Ian Cadiz	Manila Doctors Hospital	Co-PI
Philippines	Dennis Jose Sulit	Quirino Memorial Medical Center	PI
Philippines	Arturo Jr Patanao	Quirino Memorial Medical Center	Co-PI
Philippines	Candy Angelica Sigua Cabaddu	Quirino Memorial Medical Center	Co-PI
Philippines	Gregorio Rogelio	St. Lukes Medical Center	PI

Country	Name	Center	Role
Philippines	Lemuell Karla Sanchez	St. Lukes Medical Center	Co-PI
Philippines	Nannette Rey	De La Salle University & Medical Center	PI
Philippines	Julie Anne Villanueva	De La Salle University & Medical Center	Co-PI
Philippines	Kenneth Miranda	De La Salle University & Medical Center	Co-PI
Philippines	Ian Lordeo Gutierrez	De La Salle University & Medical Center	Co-PI
Philippines	Eleanor Ponayo	De La Salle University & Medical Center	Co-PI
Philippines	Lauren Kay Evangelista	De La Salle University & Medical Center	Co-PI
Philippines	Miram Timonera	Adventist Medical Centre	PI
Philippines	Celina Jo	Adventist Medical Centre	Co-PI
Philippines	Kathleen Garcia-Garingarao	Adventist Medical Centre	Co-PI
Philippines	Carlo Garingarao	Adventist Medical Centre	Co-PI
Philippines	Aireen Capitan	Adventist Medical Centre	Co-PI
Philippines	Josefina Cruz	Palawan Medical City	PI
Philippines	Armie Tabarra Cleofas	Palawan Medical City	Co-PI
Philippines	Tirador Louie	St. Paul's Hospital	PI
Philippines	Chariza Trompeta	St. Paul's Hospital	Co-PI
Philippines	Efren Jr Estoce	St. Paul's Hospital	Co-PI
Philippines	Ma Dovie Lallaine Ygpuara	St. Paul's Hospital	Co-PI
Philippines	John Dennis Alcaraz	Jabez Medical Center	PI
Philippines	Leah Alcaraz	Jabez Medical Center	Co-PI
Philippines	Maria Teresa B Abola	Philippine Heart Centre	PI
Philippines	Leahdette Padua	Philippine Heart Centre	Co-PI
Tanzania	Kajiru Kilonzo	Pamoja Tunawez Women's Centre	PI
Tanzania	E Harry Mwerinde	Pamoja Tunawez Women's Centre	PI
Tunisia	Habib Gamra	Fattouma Bourguiba University Hospital	PI
Tunisia	Majed Hassine	Fattouma Bourguiba University Hospital	Co-PI
Tunisia	Mejdi Ben Messaoud	Fattouma Bourguiba University Hospital	Co-PI
Tunisia	Marouane Mahjoub	Fattouma Bourguiba University Hospital	Co-PI

Country	Name	Center	Role
Tunisia	Ali Ben Khalfallah	MENZEL BOURGUIBA REGIONAL HOSPITAL	PI
Tunisia	Habib Haouala	MILITARY HOSPITAL OF INSTRUCTION OF TUNIS	PI
Tunisia	Abdeddayem Haggui	MILITARY HOSPITAL OF INSTRUCTION OF TUNIS	Co-PI
Tunisia	Sondes Kraiem	Habib Thameur Hospital	PI
Tunisia	Fethia Ben Moussa	Habib Thameur Hospital	Co-PI
Tunisia	Faouzi Maatouk	Fattouma Bourguiba University Hospital	PI
Tunisia	Samir Nouira	Fattouma Bourguiba University Hospital	PI
Tunisia	Wafa Zhani	Fattouma Bourguiba University Hospital	Co-PI
Tunisia	Khaoula Bouaziz	Fattouma Bourguiba University Hospital	Co-PI
Tunisia	Asma Sriha-Belguith	Fattouma Bourguiba University Hospital	PI
Tunisia	Hela Abroug	Fattouma Bourguiba University Hospital	Co-PI
Tunisia	Manel Ben Fredj	Fattouma Bourguiba University Hospital	Co-PI
